# Stakeholder perspectives on vision screening for drivers in Gauteng: Policy review implications

**DOI:** 10.4102/hsag.v30i0.2826

**Published:** 2025-02-21

**Authors:** Gloria T. Tamenti, Tuwani A. Rasengane, Khathutshelo P. Mashige

**Affiliations:** 1 Discipline of Optometry, School of Health Sciences, University of KwaZulu-Natal, Durban, South Africa; 2 Department of Optometry, Faculty of Health Sciences, University of the Free State, Bloemfontein, South Africa

**Keywords:** vision standards, driving licence, vision screening, driving licensing testing centre, site managers, driver’s licence examiners

## Abstract

**Background:**

Effective implementation of vision screening standards at driving licensing testing centres (DLTCs) necessitates adequate administrative and resource management.

**Aim:**

To ascertain the perspectives of site managers and driver’s licence examiners regarding vision screening standards at DLTCs in Gauteng province, South Africa.

**Setting:**

The study was conducted in Gauteng province, South Africa.

**Methods:**

A qualitative study that utilised interview questionnaires to assess site managers’ and driver’s licence examiners’ perspectives on the vision screening standards at the DLTC sites.

**Results:**

A total of 30 participants, comprising 15 site managers and 15 driver’s licence examiners, were interviewed from 15 out of 32 randomly selected functional DLTCs in the Gauteng province. The current vision policy and driving practices have remained unchanged since their inception. The vision screening equipment utilised at DLTCs has transitioned from manually operated to automated systems. Nevertheless, frequent machine breakdowns, primarily attributed to inadequate maintenance plans, were among the most frequently reported barriers to efficient vision screening.

**Conclusion:**

This study highlights the need to review and update vision-related policies and practices for driver licensing in South Africa. This entails establishing a Medical Advisory Board to ensure appropriate vision screening functions for driving and reliable vision screening technology. Specifically, this will include implementing a vision-related examiner’s training programme with a certificate of competence, an electronic eye-testing interface, proactive equipment maintenance programmes, improved quality control mechanisms and standardisation of the vision screening process across all DLTCs.

**Contribution:**

This study identified challenges to the effective implementation of vision screening for driving.

## Introduction

Research indicates that vision accounts for 90% of the information a driver receives, encompassing various sensory functions, including cognitive and motor functions (Alvarez-Peregrina et al. 2021; Owsley & McGwin 2010; Sivak 1996). Impaired vision is associated with poor driving performance, negatively impacting driving ability and increasing the risk of motor vehicle accidents (Nguyen et al. 2020; Wood 2022). This is because of reduced vision, visual acuity and/or visual fields secondary to uncorrected refractive errors or pathological conditions. Road safety poses a major public health challenge in most countries, including South Africa (Mpunzi 2018; Shipp et al. 2000). Consequently, vision screening has become a crucial element of road safety strategies in most countries, such as the United States, the United Kingdom and many African countries (Bron et al. 2010; De Laey & Colenbrander 2006; Yan et al. 2019). In South Africa, vision screening is mandatory for both obtaining and renewing a driver’s licence.

Vision screening is the sole assessment conducted at the Driver Licensing Testing Centre level nationwide, as mandated by Regulation 102 of the *National Road Traffic Act*, (Act 93 of 1996) (Mathebula, Boadi-Kusi & Kok 2016). Vision screening is a component of the road safety regulation designed to identify drivers with visual impairments, ensure compliance with minimum visual standards for safe driving and implement licence restrictions when necessary. Globally, most countries such as Australia, United Sates (US) and India have adopted licence restrictions as a public health measure to mitigate the risk of car accidents by focusing on individual drivers while maintaining acceptable levels of mobility, especially for drivers 65 years and older and those with visual impairments (Honavar 2022; Langford & Koppel 2011; Stutts, Stewart & Heusen-Causey 2000). The nature and extent of these licence restrictions vary between countries (Joyce et al. 2018; Watson, Leal & Soole 2013).

In South Africa, driver restrictions pertain solely to the use of spectacles or contact lenses. Consequently, licensing authorities and eyecare professionals are tasked with ensuring that drivers comply with the minimum visual requirements specified for each licence code, irrespective of whether refractive correction is used. According to Regulation 102 of the *National Road Traffic Act* (Act 93 of 1996), individuals may be disqualified from obtaining or holding a learner’s or driving licence for light motor vehicle (categories A1, A, B or EB) unless they meet a minimum visual acuity of 6/12 (20/40) in each eye, or 6/9 (20/30) in cases where the visual acuity in one eye is less than 6/12 (20/40), with or without refractive correction. Additionally, these drivers must have a minimum temporal visual field of 70 degrees temporal in one eye; if one eye has a temporal visual field of less than 70 degrees or is blind, a total horizontal visual field of at least 115 degrees is required, with or without refractive correction. For heavy motor vehicle drivers (categories C1 C, EC1 or EC), the minimum visual acuity requirement is 6/9 (20/30) in each eye, along with a temporal visual field of at least 70 degrees, with or without refractive correction.

Consequently, individuals who do not meet the established visual requirements are required to undergo vision screening conducted by a registered optometrist or ophthalmologist at their own expense. The results of the vision screening test (in the form of a vision screening certificate) must be submitted to the driving licence testing centre. Failure to provide the required certificate will result in the driving licence testing centre declining to issue a learner’s or driving licence with the specified licensing code, as stipulated in the licensing application form (South African Government 1996).

However, there are no standardised protocols concerning the frequency of reviewing the above-mentioned regulation or the uniformity of equipment used. The current study aimed to investigate the perspectives of key stakeholders on the standards of vision screening for drivers. These stakeholders included site managers and examiners of driver’s licences (EDLs) at driving licensing testing centres (DLTCs).

## Research methods and design

To address the research questions, two distinct interview schedules were developed to capture the perspectives of both DLTC managers and examiners. The objective was to understand the current phenomena comprehensively from individuals who were directly involved and possessed subject matter expertise. The initial section addressed demographic variables, while the subsequent section included questions regarding policy, vision screening procedures, licence renewal period, quality assurance and suggested changes to improve service delivery. Before data collection, a semi-structured, open-ended interview questionnaire with probe questions was tested and validated. Interviews were recorded using an audio recorder. The recordings were transcribed, deidentified and stored electronically in password-protected files. The transcripts from the in-depth interviews were analysed using a deductive thematic analysis method. Data were coded, and themes were developed, forming the basis of the analysis. Common statements were collated and reported in narratives, with specific individual participant statements presented as quotes to substantiate the expressed views (Ayukotang, Moodley & Mashige 2024).

To ensure participants’ anonymity, pseudonyms were assigned: letters A to O for DLTC managers and EA to EO for examiners. The first alphabetical letter (A to O) corresponds to the number of participants, while the second alphabetical letter (assigned to examiners) links each examiner to their respective manager for ease of analysis (Table 1 and Table 2).

**TABLE 1:** Demographics of driving licensing testing centre managers.

Participants	Gender	Title of current position	Years in current position	Name of DLTC
A	Male	Management Rep	9	Centurion
B	Female	Management Rep	5	Etwatwa
C	Male	Management Rep	11	Kagiso
D	Female	Manager	3	Kempton Park
E	Male	Acting Centre Manager	8	Mabopane
F	Female	Management Rep	10	Maponya
G	Male	Management Rep	5	Midrand
H	Male	Assistant Manager	5	Mogale City
I	Male	Management Rep	8	Randburg
J	Male	Management Rep	12	Randfontein
K	Female	Management Rep	14	Sandton
L	Male	Manager	19	Temba
M	Female	Management Rep	14	Vereeniging
N	Female	Management Rep	6	Watloo
O	Female	Centre Manager	9	Xavier

DLTC, driving licensing testing centre.

**TABLE 2:** Demographics of driving licensing testing centre examiners.

Participants	Gender	Title of current position	Years in current position	Name of DLTC
EA	Male	Management Rep	14	Centurion
EB	Female	Examiner	5	Etwatwa
EC	Female	Examiner	15	Kagiso
ED	Male	Examiner	14	Kempton Park
EE	Female	Management Rep	13	Mabopane
EF	Female	Examiner	10	Maponya
EG	Female	Examiner	10	Midrand
EH	Male	Management Rep	11	Mogale City
EI	Male	Examiner	9	Randburg
EJ	Male	Examiner	3	Randfontein
EK	Female	Examiner	19	Sandton
EL	Female	Examiner	10	Temba
EM	Female	Examiner	18	Vereeniging
EN	Female	Examiner	12	Watloo
EO	Male	Examiner	13	Xavier

DLTC, driving licensing testing centre.

### Ethical considerations

Ethical clearance was secured from the BREC of the University of KwaZulu-Natal, Durban, South Africa (BREC/00000664/2019). Written permissions were also obtained from the overseeing authorities at the various DLTC sites. The study adhered to the principles outlined in the Declaration of Helsinki for research involving human subjects. Written informed consent was obtained from all participants, who were informed of their right to withdraw from the study at any time without any consequences. The identities of all participants were anonymised to ensure confidentiality.

## Results

The interviews for managers and EDL officials were conducted between August 2021 and October 2021 across 15 DLTCs in the Gauteng province. A total of 15 managers and 15 EDL officials participated in the interviews. Among the 15 site managers, there were eight men (53.3%) and seven women (46.7%). The median work experience for the site managers was 9 years, with an interquartile range (IQR) of 5–12 years. Of the 15 EDL officials interviewed, 60% were women. The median work experience for the EDL officials was 12 years, with an IQR of 9–14 years. Table 1 and Table 2 summarise the demographic characteristics of the DLTC managers and examiners, respectively.

The collected data, including transcribed interview responses, were analysed to identify key concepts aligned with the research objectives. The researcher discerned emerging themes by allowing the data to guide the thematic development. Data were filtered and classified based on key issues, themes and concepts articulated by participants. Various codes were assigned to the data sets to elucidate the collected information. Codes with analogous meanings were consolidated. Patterns and relationships among the code groups were then examined and further interpreted.

### Theme 1: Policy

The participating DLTC managers were queried regarding their awareness of any government bill tabled for the amendment of legislation concerning the requirement of vision screening for driving. None of the managers were aware of such a bill.

Participant I stated:

‘We are not aware of any policy change or efforts to review the policy on vision screening standards.’

### Theme 2: Vision screening procedures

Participants were queried regarding any modifications to the vision screening procedures for driving over the past decade. Participants reported that changes were predominantly associated with the equipment used for testing. Specifically, respondents noted a transition from the Orthorator, which necessitated more manual operation, to more automated machines.

Participant A shared:

‘There has been an upgrade of machines but testing the same vision skills, just upgraded technology.’

Participant G reported:

‘There has been a shift, from using the Snellen charts, which only measured visual acuity, to introducing the Orthorator and now more automated machines like Live Capture Unit [*LCU*] and Live Enrollment Unit [*LEU*].’

In addition, participants were asked about the procedure they follow when an applicant fails to meet the minimum vision screening requirements for driving and is declared visually unfit to drive by an eye care practitioner. All participating DLTC managers indicated that the individual’s licence code would be downgraded to a lesser code that aligns with the vision requirements. However, applicants who do not consent to the downgraded licence code will not be issued a driving licence.

Participant I expressed:

‘Depending on the driver’s code. If the driver has a higher licence code and is supported by the eye test certificate, the driver will be downgraded. Alternatively, if the code is that of a private driver, they sign a form that their licence can be cancelled completely.’

Participant H depicted:

‘Downgrade from heavy motor vehicle to light vehicle. Officials must write a report and send it to the provincial help desk to downgrade or cancel the licence [*take it out of the system*]. If the eye condition can be corrected through operation, reinstatement can happen after an eye operation with a letter from the ophthalmologist and the eye test certificate as proof of having undergone the procedure.’

### Theme 3: Licence renewal period

Participants were surveyed to assess their views on the adequacy of vision screening conducted every 5 years, coinciding with the current licence renewal period.

Participant M indicated:

‘The general practice of eye tests at optometrists are typically done every two years. Why is the licence one different?’

Participant E reported:

‘Two and a half years may be adequate since vision can change anytime.’

### Theme 4: Quality assurance and management

Participants were surveyed about the maintenance and calibration of vision screening machines. Participants reported the lack of a monitoring or quality management system to evaluate the efficiency or accuracy of on-site vision screening. It was noted that departmental audits are conducted once or twice annually by the Road Traffic Management Corporation (RTMC) and the Department of Transport (DoT).

Participant I reported:

‘The illiterate E is blurry, and calibration is needed. There have been instances where four people would fail one after the other, but after rebooting the machine, they pass the vision tests.’

Participant O indicated:

‘It happens regularly where the letter E inside the vision screener is not visible or projecting, sometimes resolves after rebooting and other times not.’

Participant J pointed out:

‘We do not know if calibration is done because no calibration certificates are issued.’

Participants were queried regarding the limitations or shortcomings of conducting vision tests at their respective DLTCs. The predominant limitation identified by participants was the frequent breakdown or offline status of the eye test machines.

Participant EL stated:

‘These machines are mostly offline because of the network connectivity issues, and sometimes fibre is stolen.’

Participant EM indicated:

‘There were several times when these machines were offline for two to three days meaning the centre could not function.’

Participants reported that the lack of sufficient functional machines at the DLTCs negatively affects service delivery.

Participant E reported:

‘Machines are limited, we were promised six machines three years ago, and we are with only two machines.’

Participant ED expressed:

‘We are currently operating with three out of seven machines because the others are not working.’

Power outages were also identified as significant constraints in most DLTCs. Notably, only a limited number of DLTCs possess backup generators, enabling them to maintain operations during power interruptions.

Participant EG stated:

‘We experience load-shedding almost daily for 1–2 hrs at the time.’

Participant EA highlighted:

‘This centre is affected by load-shedding, cable theft and burglaries, which impacts service delivery.’

Participant EB shared:

‘When there is load-shedding, we close the centre because we cannot work for the duration of the power cuts.’

### Theme 5: Suggestions for improvement

There is a mandatory on-site vision screening at DLTC locations for individuals seeking to obtain or renew a driver’s licence. Alternatively, applicants may submit a valid eye test certificate from a registered eye care practitioner, confirming compliance with Regulation 102 of the *National Road Traffic Act* (Act 93 of 1996). However, anecdotal evidence indicates that both processes are susceptible to fraudulent activities. As such, participants were invited to propose recommendations for improving the integrity and efficiency of these processes.

Participants suggested support for discussions that are underway to introduce an electronic eye-testing interface to allow optometrists to upload or capture the eye test results directly on the electronic National Administration Traffic Information System (eNaTIS).

Participant I stated:

‘Technical committee discussions are ongoing to enable optometrists to be linked to capture eye test results directly to the eNatis system. To reduce fraudulent certificates and administration required in capturing the eye test results.’

Participant G indicated:

‘To link optometrists to the DLTC technology to be able to send the eye test report. Similar to roadworthy certificates [*paperless*].’

Participant M expressed:

‘So, integrating the eNatis system with online optometry reporting will eliminate fraud.’

Participants also proposed stationing government-employed optometrists at DLTCs.

Participant K stated:

‘Accommodating optometrists within the DLTCs will assist with fraud.’

Participant L indicated:

‘It would help if government-employed optometrists are stationed at DLTCs; we will win against fraudulent eye test certificates.’

Participants also suggested a system to evaluate customer feedback.

Participant G highlighted:

‘We are receiving complaints from applicants that they can see, but somehow they are failing with the DLTC vision screening machine. These complaints are supported by the eye test certificate indicating applicants’ visual capabilities.’

## Discussion

The study aimed to investigate the perspectives of site managers and EDLs regarding the current vision screening standards at the DLTCs in Gauteng, South Africa, and the challenges associated with their implementation. The results of this study revealed that since 1998, the minimum vision requirements for driving in South Africa have not been revised, with no indications of imminent updates. This contrasts with practices in the United Kingdom, Canada, Australia, New Zealand and the United States, where eyecare practitioners adhere to periodically reviewed and updated country-specific fitness-to-drive guidelines to enhance road safety (Allan & Gillian 2022; Dvla 2021; Lijarcio et al. 2020; Oberstein et al. 2016). In South Africa, the absence of such guidelines and a dedicated Medical Advisory Board for driver licensing may lead to ambiguous policy interpretation and application, resulting in excessive discretion by eyecare practitioners. The authors recommend the development of a clinical reference manual to guide eyecare practitioners and address vision-related ambiguities in policy, such as issues with diplopia, colour vision, contrast sensitivity and central vision loss.

Despite the absence of revisions to the vision standards for driving, the Ministry of Transport has improved vision screening efficiency over the past decade by introducing automated computer-based vision screening equipment, transitioning from the non-automated Orthorator to LCU and LEU automated vision screening devices. Managers at DLTCs reported using LEU, with some combining LEU with LCU or Orthorator. Similar transitions from non-automated to automated vision screening equipment have been observed in the US, UK, Canada, Australia and New Zealand, following comprehensive data evaluations (Straus 2005). There is, however, limited information on the relationship between clinical measures of visual function and the LCU or LEU vision screening technologies used in South African DLTCs, which may compromise the accuracy of vision tests and road safety.

Drivers presenting eye test certificates from eyecare professionals, especially those declared visually unfit, are subjected to licence downgrades or non-renewal, a practice aligned with policies in the US, Australia, Canada, New Zealand and the UK (Allan & Gillian 2022; Ko et al. 2021; Langford & Koppel 2011; Owsley & McGwin 2010; Stinchcombe et al. 2019). The study recommends implementing educational and road safety awareness programmes to highlight the importance of regular eye tests and self-assessment and to communicate the appeal process for licence downgrades or revocations. An annual vision-related training programme for examiners should be established, incorporating a written examination to assess competency and award a certificate of proficiency. Additionally, dispensing opticians should be included among the eye care professionals authorised to conduct training of DLTC examiners. This inclusion is expected to alleviate the workload of optometrists and ophthalmologists.

Examiners of driver’s licences indicated that vision screening should be performed biannually as part of licence renewal, aligning with industry recommendations for comprehensive eye tests every 2 years. This study’s findings align with recommendations from ophthalmology and optometry academies, suggesting eye examinations every 2–3 years for adults aged 20–39, every 2 years for those aged 40–60 and annually for individuals aged 61 and older (Irving et al. 2016; Taylor et al. 2004). Examiners of driver’s licences reported that vision screening machines were not maintained or calibrated and lacked a monitoring system to assess their efficiency. Proper maintenance is crucial for the quality and reliability of optical devices (Srinivasan & Thulasiraj 2003). Equipment downtime because of failures or breakdowns can lead to lost productivity and customer dissatisfaction (Nezami & Yildirim 2013). The study suggests implementing proactive maintenance programmes and better quality control mechanisms to ensure the operational efficiency and accuracy of vision screening machines. This includes training EDLs to perform basic maintenance, forming specialised repair teams and employing a computerised maintenance management system to schedule and track maintenance tasks.

Driving licensing testing centre managers agreed that identifying drivers with potential vision problems is primarily through mandatory on-site vision screening or eye test certificates from eyecare professionals. The Ministry of Transport’s plans to integrate an electronic eye-testing interface for optometry reporting into the eNatis system (RTMC 2021), similar to New York State’s Department of Motor Vehicle (DMV) Vision Registry Programme, would reduce fraudulent vision screening certificates and ensure compliance with minimum vision standards for each licence category. They also suggested on-site optometrists who would enhance the credibility of vision assessments and provide convenience to the public. Furthermore, DLTC managers suggested evaluating customer feedback to understand and improve service delivery. Implementing digital surveys, social media, WhatsApp, SMS and survey boxes could gather valuable customer feedback to enhance service delivery.

## Conclusion

This study highlights the need to modernise vision-related policies and practices in driver licensing within South Africa. Key considerations include establishing a dedicated Medical Advisory Board to oversee the implementation of appropriate vision screening protocols for driving and the integration of advanced vision screening technologies. This effort should focus on standardising all vision screening procedures, introducing an electronic eye-testing interface, ensuring proactive maintenance of equipment and enhancing quality control measures. Moreover, effective collaboration between the Ministry of Transport, Driver Licensing Testing Centres and eye care professionals is crucial to achieving these objectives. Future recommendations include having eye care professionals actively involved as part of the training programme for DLTC examiners. These include the development of an annual vision-related training programme for examiners who have completed a certificate of competency. Furthermore, it is recommended that dispensing opticians be included among the eye care professionals authorised to provide training for Driver Licensing Testing Centre examiners, perform vision screening and issue eye screening certificates. This recommendation aligns with the scope of practice of dispensing opticians and may help alleviate the workload on optometrists and ophthalmologists.
